# Neuro-ophthalmic evaluation and management of pituitary disease

**DOI:** 10.1038/s41433-024-03187-x

**Published:** 2024-07-22

**Authors:** Michael T. M. Wang, Juliette A. Meyer, Helen V. Danesh-Meyer

**Affiliations:** 1 Department of Ophthalmology, New Zealand National Eye Centre, University of Auckland, Auckland, New Zealand; 2 Vision Research Foundation, Auckland, New Zealand; 3 Auckland District Health Board, Auckland, New Zealand

**Keywords:** Brain injuries, Prognostic markers

## Abstract

Neuro-ophthalmic evaluation is a crucial component of the diagnostic and prognostic assessment of pituitary disease and compressive chiasmopathy, and can inform the timing of vision-restoring tumour resection surgery. The most common disease affecting the pituitary with neuro-ophthalmic implications are pituitary adenomas. Neuro-ophthalmic manifestations include decreased vision, abnormal colour vision and impaired visual field or diplopia. The recognition of these syndromes is critical to achieve early diagnosis and treatment and to improve prognosis. The pattern of vision loss in chiasmal compression is determined by the anatomical relationship between the pituitary lesion and optic chiasm, and potential visual field defects include bitemporal deficits, junctional scotomas, monocular cecocentral defects, and incongruous homonymous hemianopias. Rarer neuro-ophthalmic manifestations of pituitary disease include ophthalmoplegia, nystagmus, and obstructive hydrocephalus. There is growing evidence that demonstrates the strong diagnostic utility of optical coherence tomography (OCT) parameters in detecting the presence of compressive chiasmopathy, as well as the prognostic ability to predict the rate and degree of visual recovery following decompression surgery. Long-term neuro-ophthalmic monitoring is critical for detecting delayed vision loss following resection surgery, which may represent tumour recurrence or secondary complications.

## Introduction

Tumours of the pituitary gland and parasellar region comprise up to 15% of all primary intracranial lesions [1], and can be associated with reversible visual dysfunction secondary to compression of the optic chiasm in 30–70% of cases [2, 3]. Although several types of lesions can lead to compressive chiasmopathy, benign pituitary adenomas are the most commonly identified cause in adults [4]. The presentation of patients depends on the type of pituitary tumour. Functioning adenomas are more commonly diagnosed secondarily to symptoms that are associated with hormone excess, before mass effect from the tumour can cause chiasmal compression. Non-functioning pituitary adenomas generally have a greater tumour volume and more commonly present with visual impairment. Other significant compressive lesions that may cause chiasmal compression include parasellar meningiomas, craniopharyngiomas, chiasmal and third ventricular gliomas and vascular abnormalities; while astrocytomas may infiltrate the optic chiasm [2, 4].

The neuro-ophthalmic management of patients with pituitary disease encompasses understanding the spectrum of clinical symptoms and signs associated with chiasmal compression. The pattern of vision loss in compressive chiasmopathy is dependent on the anatomical relationship between the lesion and optic chiasm. The chiasm contains the decussation of nasal retinal nerve fibres while the fibres from the temporal hemiretina (nasal visual field) remain ipsilateral to enter the optic tract. Therefore, visual information corresponding to a hemifield is encoded in the contralateral optic tract. For example, the fibres that subserve the right hemifield are located in the left optic tract.

It has been historically postulated that some fibres loop anteriorly 1–2 mm into the terminal portion of the opposite optic nerve before turning to enter the chiasm, known as Willbrand’s knee. These primarily serve the superotemporal portion of the contralateral visual field from the inferonasal retinal of the contralateral eye. Therefore, damage to these fibres is the cause of junctional scotoma. It has been shown that Willebrand knee may be an artifact in monkeys, but not necessarily in humans.

**Fig. 1:**
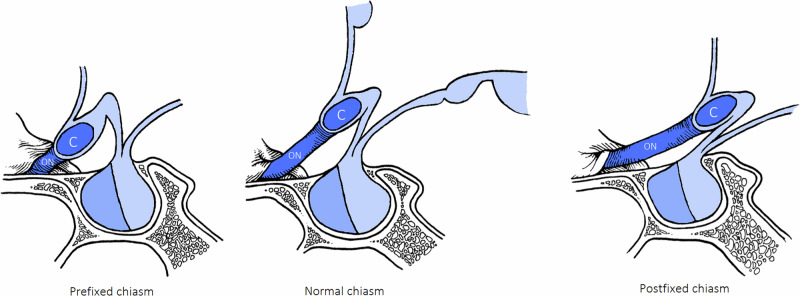
Anatomical position of optic chasm relative to the sella turcica. **A** The position of the chiasm is directly above the pituitary gland in the majority of people. A ‘prefixed’ is when the chiasm lies over the tuberculum sella while a ‘postfixed’ chiasm is when it lies over the dorsum sella.

The chiasm is located 10 mm above the sella turcica and is 12 to 18 mm in diameter. The anatomic position of the chiasm is described as prefixed, normal, or postfixed based on the relative position of the chiasm (anterior, above, or posterior) to the sella turcica. The most common position (80%) is when the chiasm overlies the diaphragma sellae (80%). Less commonly, the chiasm is prefixed, or overlying the tuberculum sellae (15%), and post-fixed, located over the dorsum sellae (5%). [Fig. 1] The chiasm itself is approximately 12–18 mm wide, 4 mm thick with a length of 8 mm (from the anterior to the posterior commissure). It is estimated that 53% of the fibres from the optic nerve decussate in the chiasm. I

Neuro-ophthalmic features associated with chiasmal compression include decreased visual acuity, visual field defects, impaired colour perception and optic atrophy. Other symptoms include diplopia, impaired stereoscopic vision and photophobia. Visual field defects associated with chiasmal compression include bitemporal hemianopic defects, junctional scotomas, monocular cecocentral defects, bitemporal hemianopic scotomas and incongruous homonymous hemianopias [5–7]. Efferent neuro-ophthalmic manifestations of pituitary and parasellar lesions, such as ophthalmoplegia and nystagmus, are less frequently observed and may be associated with cavernous sinus invasion [7, 8]. In rare cases, compression of the third ventricle can also lead to hydrocephalus [9].

In recent decades, there has been growing evidence of the powerful diagnostic and prognostic utility of spectral domain optical coherence tomography (OCT) parameters, including peripapillary retinal nerve fibre layer (pRNFL) and macular ganglion cell complex (mGCC) measurements, in identifying the presence of chiasmal compression and predicting long term visual recovery following surgical resection [10–13]. OCT is a rapid, non-contact, non-invasive ocular imaging technique based on infrared light which allows in-vivo assessment of eye tissue structure. Early diagnosis of pituitary and parasellar lesions is critical for facilitating sight-restoring decompression surgery, prior to the development of irreversible optic atrophy [2]. The purpose of the current article is therefore to highlight pertinent points in the neuro-ophthalmic evaluation and management of pituitary disease.

## Diagnosis

A comprehensive neuro-ophthalmic examination is required to detect early signs of compressive optic neuropathy before retrograde optic atrophy develops. This involves a careful history and examination encompassing visual acuity, colour vision testing, pupil responses, stereoscopic optic nerve assessment, visual field testing and ancillary tests such as optical coherence tomography.

### Clinical presentation

Clinical neuro-ophthalmic symptoms and corresponding signs of pituitary disease and/or chiasmal compression are summarised in Table 1 [2, 5–9, 14–17]. The incidence of pituitary adenomas in the United States is estimated to be approximately 2.8 per 100,000 people per year, with the peak incidence occurring in the 65–84 year group (Table 2) [18].

**Table 1:** Summary of neuro-ophthalmic signs and symptoms of pituitary disease and/or chiasmal compression [2, 5–9, 14–17].

Symptoms	Signs
Headache	
Vision loss	Normal or reduced visual acuity
• Visual blurring and/or dimming	Colour vision deficits
• Difficulty performing fine motor tasks (post-fixational blindness)	Visual field defects
	• Bitemporal field defects
	• Junctional scotoma
	• Monocular cecocentral defects
	• Incongruous homonymous hemianopia
	Optic atrophy
	• Bow-tie atrophy on ophthalmoscopy
	• Nasal and temporal sectoral pRNFL thinning, and binasal mGCC dropout on OCT scans
Binocular diplopia (paretic or hemifield slide)	Cranial nerves III, IV, and/or VI palsy
	Cranial nerve V1 and/or V2 sensory neuropathy
	Post-ganglionic Horner’s syndrome
	Decompensated phoria
Oscillopsia	See-saw nystagmus
Photophobia	
Phosphenes	
Visual hallucinations	
Hydrocephalus	
• Headache	• Papilloedema
• Nausea and vomiting	• Superior gaze palsy
• Vision loss and binocular diplopia	• Convergence retraction nystagmus
• Gait disturbance and incoordination	• Pupillary light-near dissociation
Pituitary apoplexy	
• Severe headache	• Visual field defects and/or reduced visual acuity
• Nausea and vomiting	• Cranial nerves III, IV, and/or VI palsy
• Altered consciousness	• Cranial nerve V1 and/or V2 sensory neuropathy
• Sudden vision loss and binocular diplopia	• Post-ganglionic Horner’s syndrome
	• Secondary stroke
	• Adrenal crisis

*mGCC* macular ganglion cell complex, *OCT* optical coherence tomography, *pRNFL* peripapillary retinal nerve fibre layer.

**Table 2:** Incidence of pituitary adenoma in the United States by age group [18].

Age (years)	Median (IQR) incidence per 100,000 people per year
1–17	0.19 (0.17–0.23)
18–44	2.70 (2.46–2.98)
45–64	4.49 (3.68–5.10)
65–84	6.12 (5.69–6.99)
85-	2.46 (2.22–2.88)

#### Vision loss

Examination of the afferent visual system is required in patients suspected of chiasmal compression secondary to pituitary disease. This includes visual acuity, colour vision, pupils, visual fields and optic nerve assessment. Visual acuity may be normal even in the presence of a dense bitemporal hemianopia. When visual acuity loss or reduction is present it is typically slowly progressive or detected incidentally on routine ophthalmic screening [2, 7, 19]. However, sudden visual decline can also occur with rapid expansion of the mass lesion and/or chiasmal infarction, such as in pituitary apoplexy [20, 21]. Subjective visual complaints in pituitary disease are frequently non-specific, and many patients may not perceive peripheral vision impairment [22]. Commonly reported symptoms include difficulty performing fine motor tasks requiring stereopsis and binocular vision, as well as slowly dimming and blurry vision [2, 22]. The average duration of ophthalmic symptoms at the time of diagnosis ranges from 6 to 24 months [7, 19].

Visual acuity can range from normal to no light perception in compressive chiasmopathy, and usually remain unaffected until associated field defects encroach centrally towards fixation [5, 7, 19, 23]. Colour vision deficits can be detected both in the presence or absence of reduced visual acuity [19, 24]. Patients can occasionally miss reading figures that fall in the temporal field, and temporal red desaturation may also be detected when tested across the vertical meridian [2]. Patients with complete bitemporal visual field defect will not have a relative afferent pupillary defect (RAPD). However, a RAPD will be present if there is significant asymmetry in the visual field loss.

**Fig. 2:**
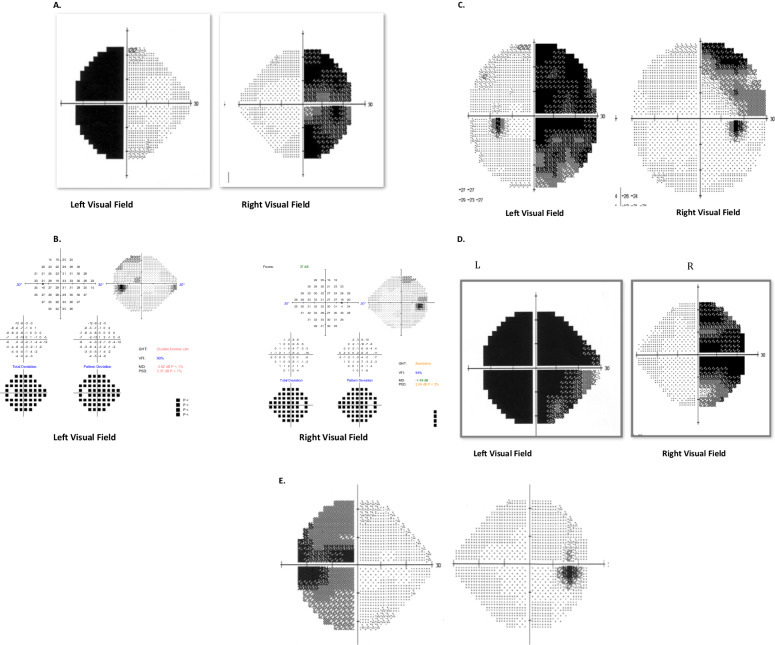
Visual Field Defects Associated With Chiasmal Compression. **A** Bitemporal hemianopia: most common lesion produced by chiasmal compression. **B** Early bitemporal loss in the superior quadrant suggestive of compression of the fibres in the inferior chiasm. **C** Right Incomplete incongruous hemianopia: pre-fixed chiasm or a tumour that impact on the optic tract. **D** Left Junctional Scotoma or ‘anterior chiasmal syndrome’: a lesion located at the medial aspect of the junction between the chiasm and the left optic nerve will affect the left optic nerve fibres and the fibres that cross in the anterior chiasm to the contralateral side producing a left central scotoma and a right temporal hemianopic defect. **E** Unilateral hemianopia: a small lesion damaging only the crossing fibres of the right eye at the anterior chiasm can produce a monocular temporal hemianopic defect.

Pituitary lesions compressing the optic chiasm cause peripheral visual field defects more commonly than compromised central acuity [5, 7, 19, 23], and therefore automated static perimetry is recommended routinely in the neuro-ophthalmic evaluation of affected patients. The pattern of visual field loss is dependent on a myriad of factors, including individual microanatomical variation in the decussation of nerve fibres, the anatomical relationship between the chiasm and skull base, as well as the type and location of the compressive lesion [2]. Chiasmal contact and compression usually do not occur until pituitary adenomas expand 8 mm or more above the diaphragma sellae [6]. Typical visual field deficits include bitemporal defects, junctional scotomas, monocular cecocentral defects, and incongruous homonymous hemianopias [2, 5–7] [Fig. 2].

Bitemporal visual field defects respecting the vertical meridian are the most commonly detected pattern in 32–81% of patients with visual impairment [5, 6, 19]. This pattern of field loss is associated with lesions affecting the central portion of the chiasm, which preferentially damage decussating fibres originating from the nasal hemiretina of both eyes [25]. In most cases, central visual acuity remains preserved [5, 7, 19, 23], although patients may miss letters on the chart in the temporal field of each eye. The bitemporal hemianopia may initially be limited to the superior quadrants in infrachiasmal lesions, such as pituitary adenomas, tuberculum sellae and medial sphenoidal ridge meningiomas, before progressing to the inferotemporal then inferonasal quadrants [7, 19]. Conversely, visual field loss associated with suprachiasmal lesions tends to begin in the inferotemporal quadrants [26]. Other causes of bitemporal visual field loss include enlarged physiological blind spots, posterior staphyloma, and refractive scotomata, although visual field defects secondary to these ocular causes do not necessarily respect the vertical meridian.

Junctional scotomas occur in 4–39% of patients with pre-operative visual impairment [5, 6, 27]. The field deficit occurs when an anteriorly located lesion compresses the medial aspect of the junction between the optic nerve and chiasm, thereby affecting nerve fibres from the ipsilateral optic nerve, as well as the Willebrand knee fibres originating from the inferonasal retina of the contralateral eye [28]. The resulting visual field defect therefore comprises an ipsilateral central scotoma and a contralateral superotemporal defect [28]. These cases highlight the importance of routine visual field assessment of the fellow eye in patients complaining of gradual monocular visual loss, as the contralateral superotemporal defect can be subtle and may not necessarily be reported subjectively.

Homonymous hemianopia and monocular defects are less commonly observed in pituitary disease [2, 29]. Homonymous hemianopic visual field deficits associated with pituitary lesions are typically incongruous, and occur when the chiasm is prefixed, or if the compressive tumour predominantly affects the lateral chiasm or optic tracts [5, 29]. Occasionally, a small lesion may only affect decussating fibres from the ipsilateral eye, and the resulting visual field deficit may be a monocular temporal hemianopia or paracentral scotoma [2, 5, 28]. Compression of the posterior portion of the optic nerve may also occur in isolation, which causes a monocular central or cecocentral defect [2, 5].

Compressive lesions may not necessarily exert the same effect on fibres originating from each eye, and asymmetrical visual field defects associated with a relative afferent pupillary defect are observed in 15–77% of patients prior to tumour resection [2, 7].

Post-fixational blindness is a visual phenomenon that can occur in patients with complete bitemporal hemianopia [30]. During convergence, the two blind temporal hemifields overlap in the region posterior to the point of fixation [2, 30]. This results in disturbances of depth perception, and difficulty in performing fine motor tasks that rely on stereopsis and binocular vision [30].

#### Optic atrophy

**Fig. 3:**
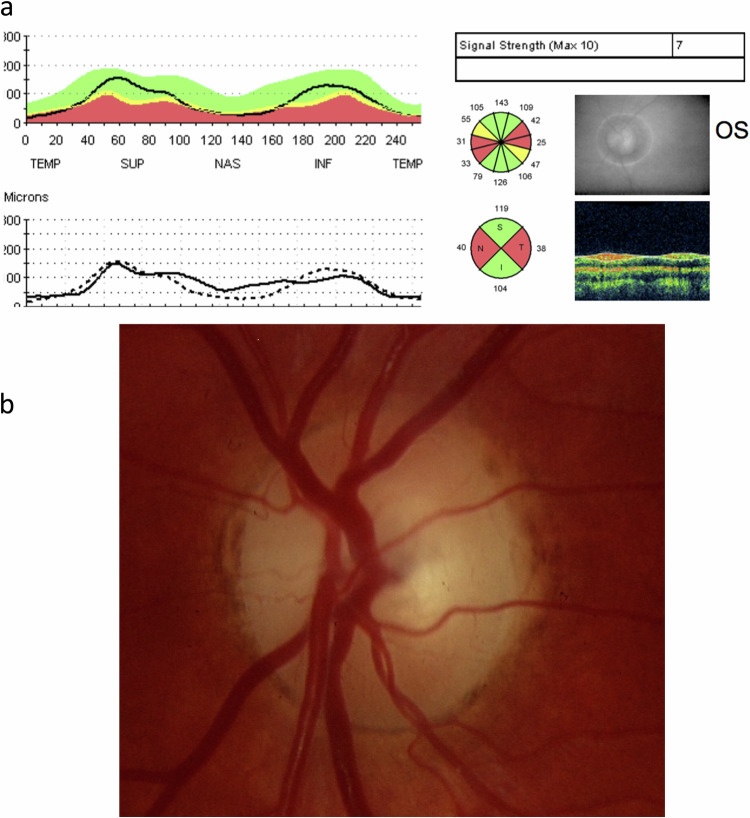
Optic nerve band atrophy. **a** Optical coherence tomography (OCT) scan of a left optic nerve in a patient who had band atrophy from chiasmal compression. Note the preferential loss of retinal nerve fibre layer nasally and temporally. **b** Optic nerve photo of the same patient.

Optic atrophy can be present or absent in chiasmal syndromes, and represents retrograde degeneration of the retinal ganglion cells and axons secondary to chronic compressive damage. In compressive chiasmopathy, decussating fibres originating from the nasal hemiretina from both eyes are preferentially affected, while temporal retinal ganglion cell fibres are relatively spared [25]. Within the retina, nasal macular fibres course directly to the temporal sector of the optic disc, while peripheral nasal fibres enter into the nasal disc sector [2]. This results in the characteristic bow-tie or band atrophy appearance visible on ophthalmoscopy, whereby pallor extends horizontally as a band across the nasal and temporal sectors of the optic disc [31] [Fig. 3].

**Fig. 4:**
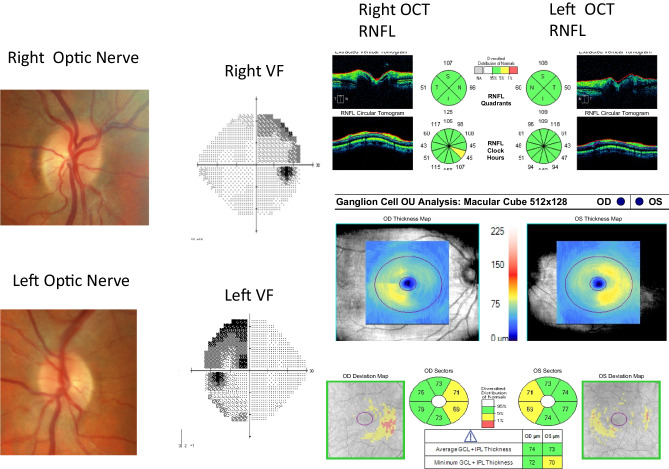
Patient with normal appearing optic nerves and OCT RNFL thickness. However, visual field test demonstrates a bitemporal hemianopia and ganglion cell layer thickness shows corresponding binasal loss.

**Fig. 5:**
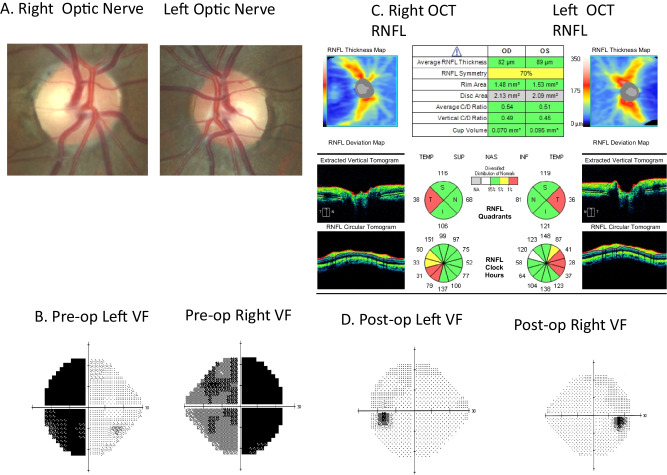
Patient with bitemporal defect. The pre-operative OCT demonstrates a RNFL thickness of 82um on the right and 89 um on the left. OCT RNFL has been shown to predict visual field recovery. Post-operative visual field shows improvement of the bitemporal visual field defect.

This pattern has also been corroborated in OCT studies. Compression of the optic chiasm caused by pituitary adenomas may induce RNFL thinning, which reflects retrograde axonal degeneration. The classic pattern caused in cases of bitemporal hemianopias is thinner nasal and temporal sectors, although diffiuse RNFL thinning also occurs [Fig. 4]. Furthermore, there is binasal mGCC atrophy in chiasmal compression, as well as preferential nasal and temporal sectoral pRNFL thinning [24, 32] [Fig. 5].

In recent years, the powerful diagnostic utility of OCT parameters has become well established, and routine mGCC and pRNFL assessment are therefore recommended in the neuro-ophthalmic evaluation of patients with suspected chiasmal compression [10–13]. Strong correlations between both OCT parameters and the severity of pre-operative visual field deficits have been consistently reported [24, 32]. However, visual field defects typically occur earlier than OCT changes, while more recent studies have suggested that mGCC dropout might precede the onset of pRNFL thinning [33]. Moreover, OCT-A studies have also demonstrated reduced peripapillary and superficial macular vessel densities in patients with chiasmal compression [34].

Pre-operative OCT pRNFL and mGCC are strong prognostic markers for predicting both the degree and rate of visual recovery following surgical resection of pituitary and parasellar lesions [10–13] Thinner pre-operative pRNFL measurements of less than 85 μm are associated with poorer long-term visual field and acuity recovery [35]. Patients with pre-operative pRNFL above the fifth percentile of age-matched normative values experience most of their visual field recovery within the first 6 weeks following surgical resection, while those with pRNFL below the fifth percentile demonstrate slower improvement during the first 6 months [11]. Several multivariate risk prediction models incorporating age, pre-operative visual function and OCT measurements, and/or MRI compression grading have also been developed, and exhibit moderate-to-high prognostic performance in predicting long-term visual recovery [10, 12].

#### Diplopia

Binocular diplopia occurs in 2–10% of patients with pituitary disease, and can develop from two main mechanisms [7, 27]. Lateral invasion of the lesion into the cavernous sinus can cause direct compression of the ocular motor cranial nerves III, IV, and VI, leading to ophthalmoplegia [30]. The resulting paretic binocular diplopia may have vertical, horizontal, or torsional components, depending on the combination of ocular motor cranial nerves affected [2, 30]. Moreover, ptosis and mydriasis might also be observed with cranial nerve III involvement. Trigeminal sensory neuropathy can occur when the ophthalmic and/or maxillary branches of cranial nerve V are affected within the cavernous sinus [2, 30]. A post-ganglionic Horner’s syndrome might be detected with damage of the oculosympathetic fibres, typically in combination with cranial nerve VI involvement [30].

Hemifield slide phenomenon is an uncommon cause of binocular diplopia in pituitary disease, and occurs in the presence of intact ocular motility [30]. In patients with bitemporal hemianopia, there is a significant reduction in overlap between the temporal hemifield of one eye from the nasal hemifield of the contralateral eye, which disrupts the ability of the brain to maintain fusion and/or control any underlying latent heterophoria [2, 30]. The resulting comitant esodeviation, exodevation, or hyperdeviation can lead to the perception of overlapping or divergent images [2].

#### Nystagmus

Seesaw torsional nystagmus is an unusual type of involuntary ocular movement, which can be associated with chiasmal compression or rostral midbrain lesions [8]. The pendular oscillations are comprised of cycles of elevation and intorsion in one eye, with synchronous depression and extorsion in the contralateral eye [2, 8]. Although the pathophysiology is not fully understood, it has been hypothesized that the loss of crossed visual input from decussating fibres at the optic chiasm can contribute to the disruption of visual-vestibular interactions [8]. In some patients, seesaw nystagmus can induce symptoms of oscillopsia [8].

#### Other neuro-ophthalmic presentations

In rare cases, postero-superior expansion of pituitary or parasellar lesions can result in compression of the third ventricle with resultant hydrocephalus from obstruction of cerebrospinal fluid flow [36]. Associated signs can include papilloedema, superior gaze palsy, convergence retraction nystagmus, and pupillary light-near dissociation [30, 36]. Hydrocephalus and papilloedema are more frequently associated with suprachiasmal than intrasellar lesions [30].

Isolated cases of compressive chiasmopathy presenting with photophobia, phosphenes, increased glare, and visual hallucinations have also been reported in the literature, although the underlying mechanisms are not fully understood [14–16].

#### Systemic symptoms

Headache is present in 33–72% of patients with pituitary lesions, and often localizes to the brow and periorbital region [37]. The symptom can arise from traction and displacement of pain-sensitive structures including the dura mater, blood vessels, and cranial nerves within the cavernous sinus [37]. Symptoms of endocrine dysfunction can be associated with both excessive secretion or deficiency of pituitary hormones, and are summarized in Table 3 [17, 38]. During pregnancy, tumour enlargement occurs in up to 30% of patients with pre-existing macroprolactinomas, which can exacerbate chiasmal compression and visual symptoms that may spontaneously improve following delivery [39].

**Table 3:** Clinical presentation of endocrine dysfunction in pituitary disease [17, 38].

Hormone	Excess	Deficiency
Prolactin (PRL)	• Amenorrhoea	
	• Galactorrhea	
	• Erectile dysfunction	
	• Gynecomastia	
	• Osteoporosis	
Growth hormone (GH)	• Enlarged hands and feet	• Fatigue
	• Frontal bossing, prognathism, enlarged nose and tongue	• Weight gain
	• Skin tags	
	• Excessive sweating	
	• Arthritis	
	• Carpal tunnel syndrome	
Adrenal corticotropic hormone (ACTH)	• Weight gain	• Fatigue
	• Round face, facial plethora	• Hypotension
	• Supra-clavicular fat	• Weight loss
	• Ecchymoses	• Nausea, vomiting, and abdominal pain
	• Muscle weakness	
	• Mood disorders	
Thyroid stimulating hormone (TSH)	• Palpitations and arrhythmias	• Weight gain
	• Weight loss	• Fatigue
	• Tremors	• Cold intolerance
	• Goitre	• Constipation
Anti-diuretic hormone (ADH)		• Diabetes insipidus

Pituitary apoplexy is an acute, life-threatening syndrome caused by infarction or haemorrhage of a pituitary tumour [17]. The condition occurs in 1.6–2.1% of patients with pre-existing pituitary tumours, and secondary haemorrhage and oedema cause rapid compression of the surrounding structures, including the optic chiasm and cavernous sinus [40, 41]. The event is usually heralded by severe headache, nausea, altered consciousness, as well as sudden deterioration in visual field defects and ophthalmoplegia [17, 40]. Extravasation of blood into the subarachnoid space can precipitate vasospasm and secondary stroke [42]. Acute endocrine dysfunction may also be associated with life-threatening complications, including adrenal crisis [43].

### Radiology and laboratory investigations

Dedicated magnetic resonance imaging (MRI) protocols, with and without gadolinium contrast, are the neuroradiological study of choice for the characterisation of intrasellar and parasellar lesions [44]. A number of studies have reported that increased tumour size on MRI is predictive of poorer visual recovery following surgical resection [10, 19, 45, 46]. High-resolution computed tomography (CT) with fine cuts between 1.5 and 3.0 mm can be considered in patients with contraindications to MRI [44].

Laboratory investigations to screen for hypopituitarism and hormonal hypersecretion are usually warranted, and should be requested in consultation with an endocrinologist [17, 47]. The clinical practice guidelines of the Endocrine Society (United States) recommend complete biochemical screening in both patients with and without symptoms of endocrine dysfunction, and are summarised in Table 4 [47].

**Table 4:** Laboratory screening tests for endocrine dysfunction in pituitary disease [47].

Screening test
Prolactin (PRL)
Thyroid stimulating hormone (TSH), thyroxine (T4)
Follicle-stimulating hormone (FSH), estradiol, testosterone
Insulin-like growth factor 1 (IGF-1), growth hormone (GH)
Adrenal corticotropic hormone (ACTH), fasting early morning cortisol

## Management

### Medical and surgical treatment

The treatment of pituitary tumours requires a multi-disciplinary approach, with medical management typically being guided by an endocrinologist, and surgical debulking performed by a neurosurgeon or otorhinolaryngologist [17]. Management decisions are dependent upon tumour size, secretory function, and associated visual impairment, and the goals of treatment include control of tumour growth, restoration of endocrine function, and preservation and/or recovery of visual function [17, 38, 48]. Although some hormone-secreting pituitary adenomas are amenable to medical treatment, most tumours with demonstrable compressive chiasmopathy and visual loss require surgical resection [17, 38, 48].

Medical treatment options can be trialled initially in prolactinomas, somatotroph and thyrotroph adenomas [49–51]. Dopamine agonists, including bromocriptine and cabergoline, have been demonstrated to reduce prolactinoma size and reverse hyperprolactinaemia [49]. In patients with macroprolactinomas, dopamine agonist therapy has been demonstrated to induce tumour shrinkage in 50–90% of cases, and normalisation of serum prolactin levels in 60–95% of cases [52]. Previous imaging studies have also reported that the greatest reduction in tumour size and serum prolactin levels occurs during the first 3–6 months of treatment, and the lack of significant improvement within this period is a strong predictor of pharmacological resistance to dopamine agonist therapy [53]. Octreotide, a somatostatin analogue, can shrink both somatotroph and thyrotroph adenomas and normalise endocrine imbalances [50, 51]. Earlier studies have shown that octreotide monotherapy can achieve biochemical control in 20–35% of patients with somatotroph adenomas, although therapeutic success can be further enhanced to 52% when combined with cabergoline treatment [54].

Pituitary tumour resection can be performed using trans-sphenoidal, trans-frontal, or trans-pterional approaches [17, 38, 48]. Surgical debulking is usually indicated in cases with confirmed compressive chiasmopathy and vision loss, cavernous sinus involvement, and pituitary apoplexy, as well as patients that are unable to tolerate or demonstrate no response to medical therapy [17, 38, 48]. In addition, prolactinomas with significant cystic features accounting for more than 80% of tumour volume on MRI T2 sequences are unlikely to respond sufficiently to medical therapy alone, and are therefore directly treated by surgical resection [17, 38, 48, 55]. Previous studies have demonstrated that visual field improvement occurs in 79–95% of patients following pituitary adenoma resection, and visual acuity improvement occurs in 45–86% of cases [2, 56]. An earlier meta-analysis of 17,509 patients showed that 5-year post-operative recurrence rates ranged from 4% in somatotroph adenomas, 11% in corticotroph adenomas, 12% in non-functioning pituitary adenomas, and 18% in prolactinomas [57]. Radiation therapy is usually administered for smaller tumours distant from the optic chiasm, or as adjunctive treatment in patients with incompletely resected tumours or post-operative recurrence [58].

### Neuro-ophthalmic surveillance

Long-term neuro-ophthalmic surveillance for pituitary and parasellar lesions is desirable for detecting delayed visual loss, which may be the first sign of tumour recurrence [59–61]. Visual acuity and colour vision testing, and automated static perimetry should ideally be performed within 2–3 months of surgical resection or radiotherapy [2, 59]. Long-term neuro-ophthalmic monitoring thereafter should ideally occur routinely at 3–12 monthly intervals, or sooner should subjective complaints of visual change be reported by the patient [2, 59]. Periodic neuroimaging studies are also warranted, although the timing might be dependent on the outcome of surgical debulking or radiotherapy response, and should be determined in consultation with the neurosurgeon and neuroradiologist [59].

Delayed vision loss following primary treatment can occur due to a variety of reasons [59, 62–64]. Chiasmal compression secondary to tumour recurrence or the expansion of fat packed within the sella turcica intra-operatively, and delayed radionecrosis may be detected on serial perimetry assessment [59, 62, 63]. Traction of the chiasm due to adhesions, or descent and herniation in secondary empty sella syndrome can also lead to post-operative vision loss [59, 64]. MRI neuroimaging is required to differentiate between the possible causes of delayed vision loss and inform ongoing treatment decisions [59, 62–64].

## Conclusions

In conclusion, neuro-ophthalmic testing forms an integral part of the diagnostic and prognostic evaluation of pituitary disease and chiasmal compression, and helps to guide treatment decisions surrounding the timing of potentially sight-preserving tumour resection surgery. The pattern of vision loss can be variable in compressive chiasmopathy and is dependent on the anatomical relationship between the lesion and optic chiasm, with potential visual field defects including bitemporal deficits, junctional scotomas, monocular cecocentral defects, and incongruous homonymous hemianopias. Less common neuro-ophthalmic presentations of pituitary lesions include ophthalmoplegia, nystagmus, and obstructive hydrocephalus. Recent studies have highlighted the strong diagnostic ability of OCT pRNFL and mGCC parameters in detecting the presence of chiasmal compression, as well as the powerful prognostic performance in predicting the rate and extent of visual recovery following tumour resection. Long-term neuro-ophthalmic surveillance is desirable for detecting delayed post-operative vision loss that can be secondary to tumour recurrence, chiasmal descent or adhesions, sellar fat expansion, and delayed radionecrosis.

## References

[1] Ostrom QT, Gittleman H, Truitt G, Boscia A, Kruchko C, Barnholtz-Sloan JS. CBTRUS statistical report: primary brain and other central nervous system tumors diagnosed in the United States in 2011-2015. Neuro Oncol. 2018;20:iv1–iv86. 30445539 10.1093/neuonc/noy131 PMC6129949

[2] Danesh-Meyer HV, Yoon JJ, Lawlor M, Savino PJ. Visual loss and recovery in chiasmal compression. Prog Retin Eye Res 2019;73:100765. 31202890 10.1016/j.preteyeres.2019.06.001

[3] Anderson D, Faber P, Marcovitz S, Hardy J, Lorenzetti D. Pituitary tumors and the ophthalmologist. Ophthalmology 1983;90:1265–70. 6664664 10.1016/S0161-6420(83)34393-1

[4] Takahashi M, Goseki T, Ishikawa H, Hiroyasu G, Hirasawa K, Shoji N. Compressive lesions of the optic chiasm: subjective symptoms and visual field Diagnostic Criteria. Neuroophthalmology 2018;42:343–8. 30524487 10.1080/01658107.2018.1438477 PMC6276958

[5] Ogra S, Nichols AD, Stylli S, Kaye AH, Savino PJ, Danesh-Meyer HV. Visual acuity and pattern of visual field loss at presentation in pituitary adenoma. J Clin Neurosci. 2014;21:735–40. 24656736 10.1016/j.jocn.2014.01.005

[6] Schmalisch K, Milian M, Schimitzek T, Lagrèze WA, Honegger J. Predictors for visual dysfunction in nonfunctioning pituitary adenomas - implications for neurosurgical management. Clin Endocrinol. 2012;77:728–34. 10.1111/j.1365-2265.2012.04457.x 22747829

[7] Poon A, McNeill P, Harper A, O’Day J. Patterns of visual loss associated with pituitary macroadenomas. Aust N. Z J Ophthalmol. 1995;23:107–15. 7546685 10.1111/j.1442-9071.1995.tb00138.x

[8] Yat-Ming Woo P, Takemura S, Ming-Yan Cheong A, Chi-Ho Chu A, Chan Y, Wong H-T, et al. Pendular Seesaw Nystagmus in a Patient With a Giant Pituitary Macroadenoma: Pathophysiology and the Role of the Accessory Optic System. J Neuroophthalmol 2018;38:65–69. 29135813 10.1097/WNO.0000000000000575

[9] Zhang D, Chen J, Li Z, Wang J, Han K, Hou L. Clinical features and management of nonfunctioning giant pituitary adenomas causing hydrocephalus. Oncotarget 2018;9:15409–17. 29632654 10.18632/oncotarget.24171 PMC5880614

[10] Lee J, Kim SW, Kim DW, Shin JY, Choi M, Oh MC, et al. Predictive model for recovery of visual field after surgery of pituitary adenoma. J Neurooncol 2016;130:155–64. 27476080 10.1007/s11060-016-2227-5

[11] Wang MTM, King J, Symons RCA, Stylli SS, Daniell MD, Savino PJ, et al. Temporal patterns of visual recovery following pituitary tumor resection: A prospective cohort study. J Clin Neurosci. 2021;86:252–9. 33775337 10.1016/j.jocn.2021.01.007

[12] Wang MTM, King J, Symons RCA, Stylli SS, Meyer J, Daniell MD, et al. Prognostic utility of optical coherence Tomography for Long-Term Visual Recovery Following Pituitary Tumor Surgery. Am J Ophthalmol. 2020;218:247–54. 32533947 10.1016/j.ajo.2020.06.004

[13] Meyer J, Diouf I, King J, Drummond K, Stylli S, Kaye A, et al. A comparison of macular ganglion cell and retinal nerve fibre layer optical coherence tomographic parameters as predictors of visual outcomes of surgery for pituitary tumours. Pituitary 2022;25:563–72. 35552990 10.1007/s11102-022-01228-w

[14] Jefferis JM, Innes WA, Hickman SJ. The presenting visual symptoms of optic chiasmal disease. Eur J Ophthalmol. 2023;33:9–20. 36147020 10.1177/11206721221125264

[15] Kawasaki A, Purvin VA. Photophobia as the presenting visual symptom of chiasmal compression. J Neuroophthalmol. 2002;22:3–8. 11937897 10.1097/00041327-200203000-00002

[16] Ram Z, Findler G, Gutman I, Tadmor R, Sahar A. Visual hallucinations associated with pituitary adenoma. Neurosurgery 1987;20:292–6. 3561738 10.1227/00006123-198702000-00016

[17] Tritos NA, Miller KK. Diagnosis and management of pituitary adenomas: a review. JAMA 2023;329:1386–98. 37097352 10.1001/jama.2023.5444

[18] Ghaffari-Rafi A, Mehdizadeh R, Ghaffari-Rafi S, Castillo JA Jr, Rodriguez-Beato FY, Leon-Rojas J. Demographic and socioeconomic disparities of pituitary adenomas and carcinomas in the United States. J Clin Neurosci. 2022;98:96–103. 35151063 10.1016/j.jocn.2022.01.032

[19] Barzaghi LR, Medone M, Losa M, Bianchi S, Giovanelli M, Mortini P. Prognostic factors of visual field improvement after trans-sphenoidal approach for pituitary macroadenomas: review of the literature and analysis by quantitative method. Neurosurg Rev. 2012;35:369–78. 22080165 10.1007/s10143-011-0365-y

[20] Seaman SC, Dougherty MC, Zanaty M, Bruch LA, Graham SM, Greenlee JDW. Visual and hormone outcomes in pituitary apoplexy: results of a single surgeon, single institution 15-year retrospective review and pooled data analysis. J Neurol Surg B Skull Base 2021;82:392–400. 35573926 10.1055/s-0040-1713104 PMC9100448

[21] Almeida JP, Sanchez MM, Karekezi C, Warsi N, Fernández-Gajardo R, Panwar J, et al. Pituitary apoplexy: results of surgical and conservative management clinical series and review of the literature. World Neurosurg. 2019;130:e988–e999. 31302273 10.1016/j.wneu.2019.07.055

[22] McDonald WI. The symptomatology of tumours of the anterior visual pathways. Can J Neurol Sci. 1982;9:381–90. 7151021 10.1017/S0317167100044280

[23] Gnanalingham KK, Bhattacharjee S, Pennington R, Ng J, Mendoza N. The time course of visual field recovery following transphenoidal surgery for pituitary adenomas: predictive factors for a good outcome. J Neurol Neurosurg Psychiatry. 2005;76:415–9. 15716538 10.1136/jnnp.2004.035576 PMC1739567

[24] Danesh-Meyer HV, Carroll SC, Foroozan R, Savino PJ, Fan J, Jiang Y, et al. Relationship between retinal nerve fiber layer and visual field sensitivity as measured by optical coherence tomography in chiasmal compression. Invest Ophthalmol Vis Sci. 2006;47:4827–35. 17065494 10.1167/iovs.06-0327

[25] Kosmorsky GS, Dupps WJ Jr, Drake RL. Nonuniform pressure generation in the optic chiasm may explain bitemporal hemianopsia. Ophthalmology 2008;115:560–5. 18082887 10.1016/j.ophtha.2007.07.004

[26] Chen C, Okera S, Davies PE, Selva D, Crompton JL. Craniopharyngioma: a review of long-term visual outcome. Clin Exp Ophthalmol. 2003;31:220–8. 12786772 10.1046/j.1442-9071.2003.00648.x

[27] Cohen AR, Cooper PR, Kupersmith MJ, Flamm ES, Ransohoff J. Visual recovery after transsphenoidal removal of pituitary adenomas. Neurosurgery 1985;17:446–52. 4047355 10.1227/00006123-198509000-00008

[28] Karanjia N, Jacobson DM. Compression of the prechiasmatic optic nerve produces a junctional scotoma. Am J Ophthalmol. 1999;128:256–8. 10458197 10.1016/S0002-9394(99)00084-7

[29] Glisson CC. Visual loss due to optic chiasm and retrochiasmal visual pathway lesions. Continuum. 2014;20:907–21. 25099100 10.1212/01.CON.0000453312.37143.d2 PMC10564022

[30] Trevino R. Chiasmal syndrome. J Am Optom Assoc. 1995;66:559–75. 7490416

[31] Monteiro MLR, Medeiros FA, Ostroscki MR. Quantitative analysis of axonal loss in band atrophy of the optic nerve using scanning laser polarimetry. Br J Ophthalmol. 2003;87:32–37. 12488259 10.1136/bjo.87.1.32 PMC1771480

[32] Tieger MG, Hedges TR 3rd, Ho J, Erlich-Malona NK, Vuong LN, Athappilly GK, et al. Ganglion cell complex loss in chiasmal compression by brain tumors. J Neuroophthalmol. 2017;37:7–12. 28192385 10.1097/WNO.0000000000000424 PMC6033516

[33] Blanch RJ, Micieli JA, Oyesiku NM, Newman NJ, Biousse V. Optical coherence tomography retinal ganglion cell complex analysis for the detection of early chiasmal compression. Pituitary 2018;21:515–23. 30097827 10.1007/s11102-018-0906-2

[34] Dallorto L, Lavia C, Jeannerot A-L, Shor N, Jublanc C, Boch A-L, et al. Retinal microvasculature in pituitary adenoma patients: is optical coherence tomography angiography useful? Acta Ophthalmol. 2020;98:e585–e592. 31808290 10.1111/aos.14322

[35] Danesh-Meyer HV, Papchenko T, Savino PJ, Law A, Evans J, Gamble GD. In vivo retinal nerve fiber layer thickness measured by optical coherence tomography predicts visual recovery after surgery for parachiasmal tumors. Invest Ophthalmol Vis Sci. 2008;49:1879–85. 18263812 10.1167/iovs.07-1127

[36] Verhelst J, Berwaerts J, Abs R, Dua G, Van Den Weyngaert D, Mahler C. Obstructive hydrocephalus as complication of a giant nonfunctioning pituitary adenoma: therapeutical approach. Acta Clin Belg. 1998;53:47–52. 9562706 10.1080/17843286.1998.11754141

[37] Gondim JA, de Almeida JPC, de Albuquerque LAF, Schops M, Gomes E, Ferraz T. Headache associated with pituitary tumors. J Headache Pain. 2009;10:15–20. 19067118 10.1007/s10194-008-0084-0 PMC3451766

[38] Melmed S. Pituitary-tumor endocrinopathies. N. Engl J Med. 2020;382:937–50. 32130815 10.1056/NEJMra1810772

[39] Luger A, Broersen LHA, Biermasz NR, Biller BMK, Buchfelder M, Chanson P, et al. ESE Clinical Practice Guideline on functioning and nonfunctioning pituitary adenomas in pregnancy. Eur J Endocrinol. 2021;185:G1–G33. 34425558 10.1530/EJE-21-0462

[40] Möller-Goede DL, Brändle M, Landau K, Bernays RL, Schmid C. Pituitary apoplexy: re-evaluation of risk factors for bleeding into pituitary adenomas and impact on outcome. Eur J Endocrinol. 2011;164:37–43. 20926593 10.1530/EJE-10-0651

[41] Sanno N, Oyama K, ’ichi, Tahara S, Teramoto A, Kato Y. A survey of pituitary incidentaloma in Japan. Eur J Endocrinol. 2003;149:123–7. 12887289 10.1530/eje.0.1490123

[42] Alsayadi S, Ochoa-Sanchez R, Moldovan ID, Alkherayf F. Cerebral vasospasm as a consequence of pituitary apoplexy: illustrative case. J *Neurosurg Case Lessons*. 2023; **5**. Available at: 10.3171/CASE22349. 10.3171/CASE22349 PMC10550606 36794733

[43] Mattke AF, Vender JR, Anstadt MR. Pituitary apoplexy presenting as Addisonian crisis after coronary artery bypass grafting. Tex Heart Inst J. 2002;29:193–9. 12224722 PMC124758

[44] MacFarlane J, Bashari WA, Senanayake R, Gillett D, van der Meulen M, Powlson AS, et al. Advances in the Imaging of Pituitary Tumors. Endocrinol Metab Clin North Am. 2020;49:357–73. 32741476 10.1016/j.ecl.2020.06.002

[45] Monteiro MLR, Zambon BK, Cunha LP. Predictive factors for the development of visual loss in patients with pituitary macroadenomas and for visual recovery after optic pathway decompression. Can J Ophthalmol. 2010;45:404–8. 20648089 10.3129/i09-276

[46] Anik I, Anik Y, Cabuk B, Caklili M, Pirhan D, Ozturk O, et al. Visual outcome of an endoscopic endonasal transsphenoidal approach in pituitary macroadenomas: quantitative assessment with diffusion tensor imaging early and long-term results. World Neurosurg. 2018;112:e691–e701. 29408649 10.1016/j.wneu.2018.01.134

[47] Freda PU, Beckers AM, Katznelson L, Molitch ME, Montori VM, Post KD, et al. Pituitary incidentaloma: an endocrine society clinical practice guideline. J Clin Endocrinol Metab. 2011;96:894–904. 21474686 10.1210/jc.2010-1048 PMC5393422

[48] Varlamov EV, McCartney S, Fleseriu M. Functioning pituitary adenomas - current treatment options and emerging medical therapies. Eur Endocrinol. 2019;15:30–40. 31244908 10.17925/EE.2019.15.1.30 PMC6587904

[49] Fachi MM, de Deus Bueno L, de Oliveira DC, da Silva LL, Bonetti AF. Efficacy and safety in the treatment of hyperprolactinemia: a systematic review and network meta-analysis. J Clin Pharm Ther. 2021;46:1549–56. 34137053 10.1111/jcpt.13460

[50] Colao A, Auriemma RS, Pivonello R. The Effects of Somatostatin Analogue Therapy on Pituitary Tumor Volume in Patients with Acromegaly. Springer Healthcare Ibérica S.L.; 2016. 10.1007/s11102-015-0677-y PMC4799266 26290466

[51] Fukuhara N, Horiguchi K, Nishioka H, Suzuki H, Takeshita A, Takeuchi Y, et al. Short-term preoperative octreotide treatment for TSH-secreting pituitary adenoma. Endocr J 2015;62:21–27. 25273395 10.1507/endocrj.EJ14-0118

[52] De Sousa SMC. Dopamine agonist therapy for prolactinomas: do we need to rethink the place of surgery in prolactinoma management?. Endocr Oncol. 2022;2:R31–R50. 37435462 10.1530/EO-21-0038 PMC10259306

[53] Petersenn S, Fleseriu M, Casanueva FF, Giustina A, Biermasz N, Biller BMK, et al. Diagnosis and management of prolactin-secreting pituitary adenomas: a pituitary society international consensus statement. Nat Rev Endocrinol 2023;19:722–40. 37670148 10.1038/s41574-023-00886-5

[54] Molitch ME. Diagnosis and treatment of pituitary adenomas: a review. JAMA 2017;317:516–24. 28170483 10.1001/jama.2016.19699

[55] Varlamov EV, Hinojosa-Amaya JM, Fleseriu M. Magnetic resonance imaging in the management of prolactinomas; a review of the evidence. Pituitary. 2020;23:16–26. 31659622 10.1007/s11102-019-01001-6

[56] Uy B, Wilson B, Kim WJ, Prashant G, Bergsneider M. Visual outcomes after pituitary surgery. Neurosurg Clin N Am. 2019;30:483–9. 31471055 10.1016/j.nec.2019.06.002

[57] Roelfsema F, Biermasz NR, Pereira AM. Clinical factors involved in the recurrence of pituitary adenomas after surgical remission: a structured review and meta-analysis. Pituitary 2012;15:71–83. 21918830 10.1007/s11102-011-0347-7 PMC3296023

[58] Minniti G, Flickinger J, Tolu B, Paolini S. Management of nonfunctioning pituitary tumors: radiotherapy. Pituitary 2018;21:154–61. 29372392 10.1007/s11102-018-0868-4

[59] Ziu M, Dunn IF, Hess C, Fleseriu M, Bodach ME, Tumialan LM, et al. Congress of neurological surgeons systematic review and evidence-based guideline on posttreatment follow-up evaluation of patients with nonfunctioning pituitary adenomas. Neurosurgery 2016;79:E541–3. 27635964 10.1227/NEU.0000000000001392

[60] Prete A, Corsello SM, Salvatori R. Current best practice in the management of patients after pituitary surgery. Ther Adv Endocrinol Metab 2017;8:33–48. 28377801 10.1177/2042018816687240 PMC5363454

[61] Cortet-Rudelli C, Bonneville J-F, Borson-Chazot F, Clavier L, Coche Dequéant B, Desailloud R, et al. Post-surgical management of non-functioning pituitary adenoma. Ann Endocrinol. 2015;76:228–38. 10.1016/j.ando.2015.04.003 26116412

[62] Esposito D, Olsson DS, Ragnarsson O, Buchfelder M, Skoglund T, Johannsson G. Non-functioning pituitary adenomas: indications for pituitary surgery and post-surgical management. Pituitary 2019;22:422–34. 31011999 10.1007/s11102-019-00960-0 PMC6647426

[63] Tachibana O, Yamaguchi N, Yamashima T, Yamashita J. Radiation necrosis of the optic chiasm, optic tract, hypothalamus, and upper pons after radiotherapy for pituitary adenoma, detected by gadolinium-enhanced, T1-weighted magnetic resonance imaging: case report. Neurosurgery 1990;27:640–3. 2234373 10.1227/00006123-199010000-00025

[64] Tabak M, Pelsma ICM, Kruit MC, van Furth WR, Biermasz NR, Notting IC. Chiasmal herniation following treatment of pituitary macroadenoma. Pituitary 2021;24:68–78. 33057947 10.1007/s11102-020-01088-2 PMC7864822

